# Dysbiosis of the gut microbiota and its effect on α-synuclein and prion protein misfolding: consequences for neurodegeneration

**DOI:** 10.3389/fcimb.2024.1348279

**Published:** 2024-02-16

**Authors:** Nasir Uddin Mahbub, Md Minarul Islam, Seong-Tshool Hong, Hea-Jong Chung

**Affiliations:** ^1^ Department of Biomedical Sciences and Institute for Medical Science, Jeonbuk National University Medical School, Jeonju, Republic of Korea; ^2^ Gwangju Center, Korea Basic Science Institute, Gwangju, Republic of Korea

**Keywords:** prion disease, prion protein, gut microbiota, short-chain fatty acids, Parkinson’s disease, α-synuclein, neuro-inflammation, and neurodegeneration

## Abstract

Abnormal behavior of α-synuclein and prion proteins is the hallmark of Parkinson’s disease (PD) and prion illnesses, respectively, being complex neurological disorders. A primary cause of protein aggregation, brain injury, and cognitive loss in prion illnesses is the misfolding of normal cellular prion proteins (PrP^C^) into an infectious form (PrP^Sc^). Aggregation of α-synuclein causes disruptions in cellular processes in Parkinson’s disease (PD), leading to loss of dopamine-producing neurons and motor symptoms. Alteration in the composition or activity of gut microbes may weaken the intestinal barrier and make it possible for prions to go from the gut to the brain. The gut-brain axis is linked to neuroinflammation; the metabolites produced by the gut microbiota affect the aggregation of α-synuclein, regulate inflammation and immunological responses, and may influence the course of the disease and neurotoxicity of proteins, even if their primary targets are distinct proteins. This thorough analysis explores the complex interactions that exist between the gut microbiota and neurodegenerative illnesses, particularly Parkinson’s disease (PD) and prion disorders. The involvement of the gut microbiota, a complex collection of bacteria, archaea, fungi, viruses etc., in various neurological illnesses is becoming increasingly recognized. The gut microbiome influences neuroinflammation, neurotransmitter synthesis, mitochondrial function, and intestinal barrier integrity through the gut-brain axis, which contributes to the development and progression of disease. The review delves into the molecular mechanisms that underlie these relationships, emphasizing the effects of microbial metabolites such as bacterial lipopolysaccharides (LPS), and short-chain fatty acids (SCFAs) in regulating brain functioning. Additionally, it looks at how environmental influences and dietary decisions affect the gut microbiome and whether they could be risk factors for neurodegenerative illnesses. This study concludes by highlighting the critical role that the gut microbiota plays in the development of Parkinson’s disease (PD) and prion disease. It also provides a promising direction for future research and possible treatment approaches. People afflicted by these difficult ailments may find hope in new preventive and therapeutic approaches if the role of the gut microbiota in these diseases is better understood.

## Introduction

Protein misfolding and aggregation are salient markers of the pathogenesis of prion and Parkinson’s diseases. Abnormal prion protein conversion propels prion diseases, while α-synuclein accumulation leads to dopamine neuron loss and motor symptoms in Parkinson’s disease (Chen et al., 2021; Srinivasan et al., 2021).

Prion diseases, marked by the intricate misfolding and aggregation of typical cellular prion proteins, result in neurodegeneration. The pivotal factor in their development is the conversion of the normal cellular prion protein (PrP^C^) into an abnormal isoform (PrP^Sc^) (Westergard et al., 2007; Atkinson et al., 2016). The unambiguous triggers for this transformation are not fully understood, but it is believed to involve the interplay between PrP^C^ and existing PrP^Sc^ molecules or other factors that endorse the misfolding process. The misfolded PrP^Sc^ isoform can act as a template and induce the conversion of normal PrP^C^ into the abnormal form (Zhou and Xiao, 2013; Atkinson et al., 2016; Liu et al., 2017). The accumulation of PrP^Sc^ disrupts normal cellular functions and contributes to the evolution of neurodegenerative processes (Westergard et al., 2007). The mechanisms by which PrP^Sc^ propagates, and spreads are enigmatic but may involve cell-to-cell transmission, release, and uptake of PrP^Sc^ aggregates, and the involvement of specific brain regions or cell types. It can cause mitochondrial dysfunction, oxidative stress, impaired protein clearance mechanisms, and disruption of synaptic communication (Picca et al., 2020). These pathological modifications contribute to neuronal degeneration and cell death. This inflammatory response is thought to contribute to the progression of neurodegeneration and can further aggravate the pathological processes. As the neurodegenerative processes progress, the clinical manifestations of prion diseases come out. These may comprise cognitive impairment, behavioral changes, motor dysfunction, and neurological symptoms specific to the prion disease subtype.

Parkinson’s, analogous to prion disease involves a complex interplay between genetic, environmental, and cellular factors, neuronal dysfunction, and neurodegeneration (Brundin et al., 2016; Meade et al., 2019; Jan et al., 2021).The pathogenesis of Parkinson’s disease is portrayed by the atypical aggregation of a protein called α-synuclein usually exists in a folded state within neurons (Iwatsubo, 2003; Stefanis, 2012; Burre, 2015). However, in Parkinson’s disease, α-synuclein misfolds and induces Lewy bodies and Lewy neurites (Breydo et al., 2012; Gomez-Benito et al., 2020; Calabresi et al., 2023). The substantia nigra is responsible for producing dopamine, a neurotransmitter indispensable for coordinating movement and α-synuclein aggregates can deteriorate mitochondrial function within neurons (Stefanis, 2012; Mor et al., 2019; Gomez-Benito et al., 2020; Jeon et al., 2020; Minakaki et al., 2020). Mitochondria are accountable for generating energy in cells, and their dysfunction contributes to oxidative stress and cellular damage, ultimately leading to neuronal degeneration (Guo et al., 2013). In addition, α-synuclein aggregates and impaired mitochondrial function lead to an imbalance between the production and clearance of reactive oxygen species (ROS) within cells (Esteves et al., 2011; Zaltieri et al., 2015; Lin et al., 2019; He et al., 2020). Excessive ROS production leads to oxidative stress, causing further detriment to neurons and exacerbating the pathogenesis of Parkinson’s disease (Zaltieri et al., 2015). On the other hand, the normal evacuating mechanisms responsible for removing misfolded or damaged proteins, such as the ubiquitin-proteasome system and autophagy, are impaired in Parkinson’s disease (Ebrahimi-Fakhari et al., 2012; Fecto et al., 2014). This defect leads to the accumulation of α-synuclein aggregates and other toxic protein species within neurons. Further, Parkinson’s disease is affiliated with neuroinflammation, characterized by the triggering of immune cells called microglia and the release of pro-inflammatory molecules in the brain. Neuroinflammation tends to the progression of neuronal destruction and degeneration in Parkinson’s disease (Muzio et al., 2021; Araujo et al., 2022; Isik et al., 2023; Zhang et al., 2023). The α-synuclein aggregates can propagate from one neuron to another, potentially contributing to the progression of Parkinson’s disease throughout the brain (Gomez-Benito et al., 2020; Hijaz and Volpicelli-Daley, 2020). This scattering process may occur through a prion-like mechanism (Oueslati et al., 2014), where misfolded α-synuclein is transmitted from neuron to neuron, escalating the pathological changes. Emerging evidence suggests that gut microbiota participates in neurodegenerative diseases, including prion diseases and Parkinson’s disease (Smith et al., 2017; Shu et al., 2023; Sleutel et al., 2023).

Meanwhile, misfolded prions can interact with bacterial Curli proteins which perform as a template for amyloid fibril formation through a cross-seeding event, leading to the inception and propagation of protein misfolding and aggregation insinuating a potential link between the gut microbiome and prion pathogenesis (Smith et al., 2017; Sleutel et al., 2023). The gut microbiome can affect the integrity of the intestinal barrier, and disruptions in this barrier have been witnessed in prion-infected animals (Yang et al., 2020; Kushwaha et al., 2023; Shu et al., 2023). It has been propounding that alterations in the gut microbiota composition or function could affect the permeability of the gut barrier, allowing the translocation of prions from the gut to the brain (Kujala et al., 2011; Donaldson et al., 2012; Giau et al., 2018).

In recent years, mounting evidence has accentuated the relevance of the gut-brain axis and gut microbiome in Parkinson’s disease (PD) development and advancement (Caputi and Giron, 2018; Menozzi et al., 2021; Nielsen et al., 2021; Tan et al., 2022; Li et al., 2023). The theory gaining traction posits that PD could emanate in the gut, with subsequent progression into the brain, possibly accelerated by the interconnectedness of enteric neurons in the gastrointestinal wall and the central nervous system (Carabotti et al., 2015; Geng et al., 2022). Extrinsic stressors are thought to initiate an immune response in the gut (Bajinka et al., 2020), which may stimulate and disseminate pathology from the enteric system to the brain through the vagal nerve. Research has revealed that individuals with Parkinson’s disease (PD) exhibit apparent alterations in their gut microbiome compared to healthy individuals (Gorecki et al., 2019; Salim et al., 2023). These changes involve shifts in the heterogeneity and composition of the gut microbial community. Animal studies further demonstrate that certain gut bacteria can produce metabolites that influence the aggregation of α-synuclein, a protein linked to PD pathology in the brain (Fitzgerald et al., 2019; Lei et al., 2021; Yan et al., 2021; Zhu et al., 2022). Preclinical investigations have evinced that manipulating the gut microbiota through probiotics, antibiotics, or fecal microbiota transplantation can impact motor symptoms and pathology in animal models of PD (Zhu et al., 2022; Salim et al., 2023).

## Decipher the contribution of the gut microbiome in prion disease progression

**Figure 1:**
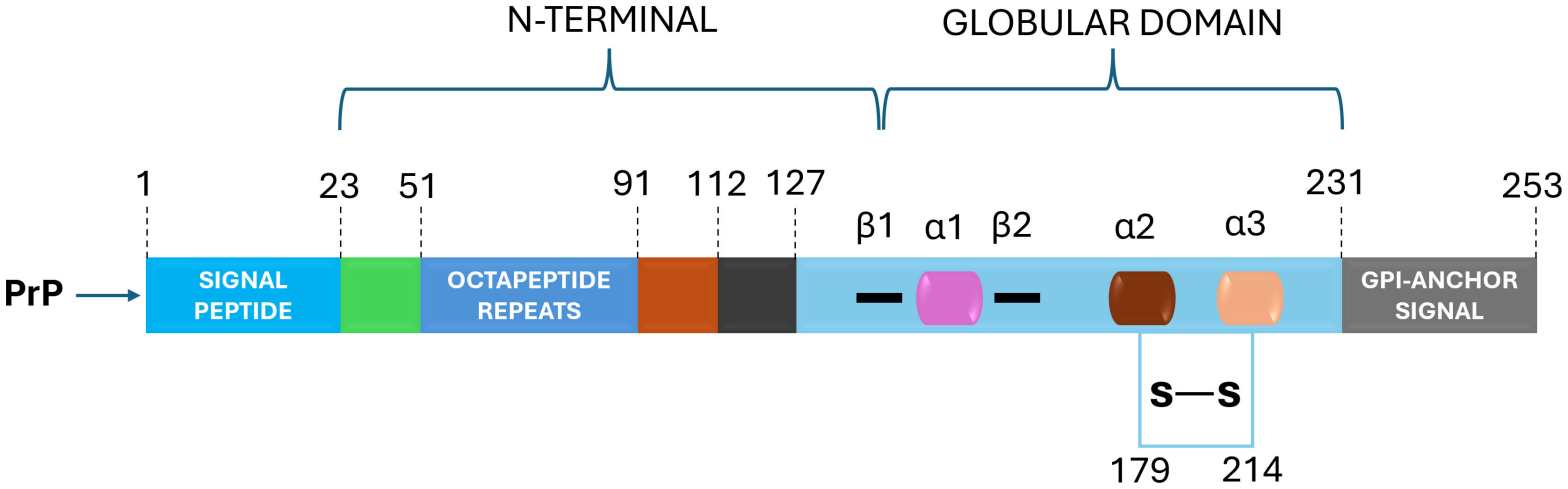
PrP^C^ Structural Domains: Navigating Prion Protein's Complexity. The prion protein (PrP^C^) is characterized by distinct domains – a disordered N-terminal with a pivotal charged region for endocytosis, octapeptide repeats binding metal cations and a hydrophobic tract. The C-terminal boasts α-helices and β-strands, hosting post-translational modifications: N-glycans enhance function, a disulfide bridge bolsters structure and a C-terminal GPI anchor affixes PrP^C^ to the plasma membrane. This intricate architecture defines PrP^C^'s multifunctional nature.

The prion protein, or PrP^C^, is a cellular glycoprotein found in the membranes of neurons and other cells (Westergard et al., 2007; Castle and Gill, 2017; Miranzadeh Mahabadi and Taghibiglou, 2020). PrP^C^ is predominantly alpha-helical in structure and plays a role in various cellular functions (Spielhaupter and Schatzl, 2001; Castle and Gill, 2017; Miranzadeh Mahabadi and Taghibiglou, 2020; Cha and Kim, 2023). In prion diseases, the PrP^C^ undergoes a conformational change, leading to the formation of the pathogenic form, known as PrP^Sc^ (scrapie PrP) (Nicholson et al., 2002; Bae et al., 2009; Baral et al., 2019). The mature prion protein features an N-terminal, unfolded domain, and a C-terminal, globular domain with three α-helices and a small, two-stranded β-sheet (**Figure 1**) (Zahn et al., 2000; Requena and Wille, 2017; Daude et al., 2022). In contrast, PrP^Sc^ is enriched in β-structure and forms multiple quaternary structures, including oligomers, amorphous aggregates, amyloid fibrils, and two-dimensional crystals (Requena and Wille, 2017; Yamaguchi and Kuwata, 2018). PrP^Sc^ is rich in beta-sheet structures and tends to aggregate into insoluble amyloid fibrils (Corsaro et al., 2012; Torrent and Lange, 2012; Zhang and Zhang, 2013; Diociaiuti et al., 2021; Willbold et al., 2021). These aggregates can accumulate in the brain and lead to neurodegeneration. The abnormal folding of PrP^C^ into a crucial stage in the genesis of prion pathologies is PrP^Sc^, as it can trigger a chain reaction, converting other normal PrP^C^ molecules into the pathogenic form (Zhou and Xiao, 2013; Hackl and Becker, 2019). This self-propagating process is the basis for the transmissible nature of prion diseases.

Currently, two primary mechanistic models have been advanced to explain the self-propagation of PrP^Sc^ from newly synthesized PrP^C^ and its eventual aggregation into amyloid fibrils: the template-assisted model (a) and the nucleation-polymerization model (b) (Abid and Soto, 2006; Corsaro et al., 2012; Sun et al., 2023).

**Figure 2:**
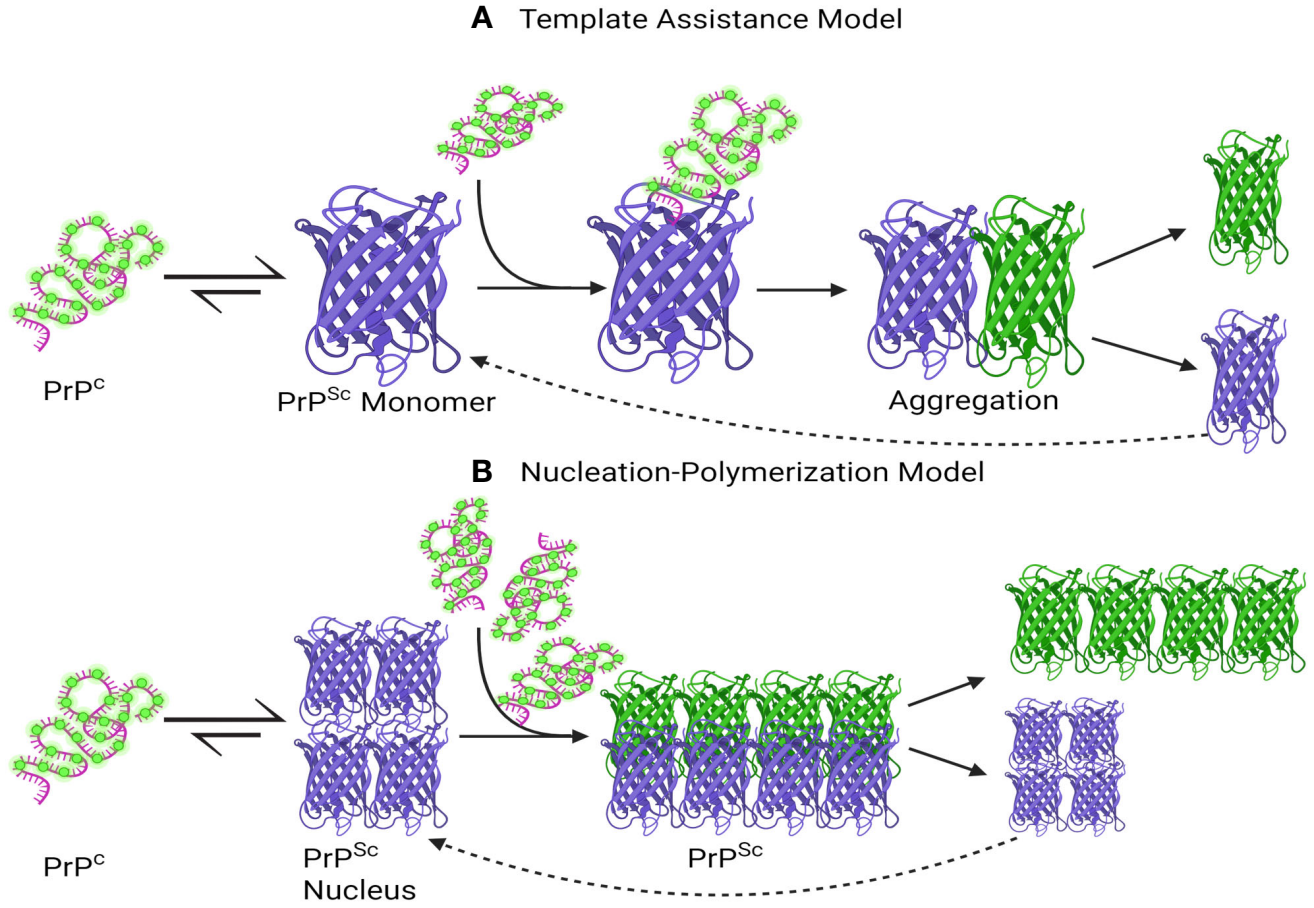
A schematic depiction has been formulated to elucidate the proposed mechanisms underlying the conversion of PrP^C^ into PrP^Sc^ and the subsequent process of aggregation. **(A)** Template assistance Model, **(B)** Nucleation-Polymerization Model. The interaction between PrP^Sc^ and PrP^C^ is depicted in the Template Assistance Model **(A)**, where PrPSc functions as a template and causes conformational change in PrP^C^. The spread of prion disease depends on this template-assisted conversion, which triggers aggregation later. On the other hand, a multi-step nucleation and polymerization process is depicted in the Nucleation-Polymerization Model **(B)**. The first step is the formation of a nucleus, or seed, which catalyzes the transformation of PrP^C^ molecules into PrP^Sc^ and then polymerization. To convert PrP^C^ to PrP^Sc^, both models highlight the importance of templating and sequential conformational changes, with subsequent aggregation events contributing to the progression of prion diseases. This visual representation aids in elucidating the intricate molecular events central to prion pathology. Created with BioRender.com.

In the template-assisted model (a), this process entails the interaction between exogenously introduced (or spontaneously generated) PrP^Sc^ molecules and endogenous PrP^C^. This interaction serves as a catalyst for the conversion of PrP^C^ into PrP^Sc^, ultimately leading to the formation of a stable oligomeric aggregate (Corsaro et al., 2012). Within the nucleation-polymerization model (b), the progression of proper folding in newly synthesized prion peptides traverses various intermediate stages. A subset of these intermediates possesses the inherent capacity for self-association, culminating in the formation of non-native oligomeric species distinguished by their diverse sizes and structural characteristics. In the presence of these stable oligomeric aggregates, there exists a notable propensity for the conversion of PrP^C^ into PrP^Sc^, as delineated by the tenets of the model (**Figure 2**) (Zhong, 2010; Corsaro et al., 2012).

**Figure 3:**
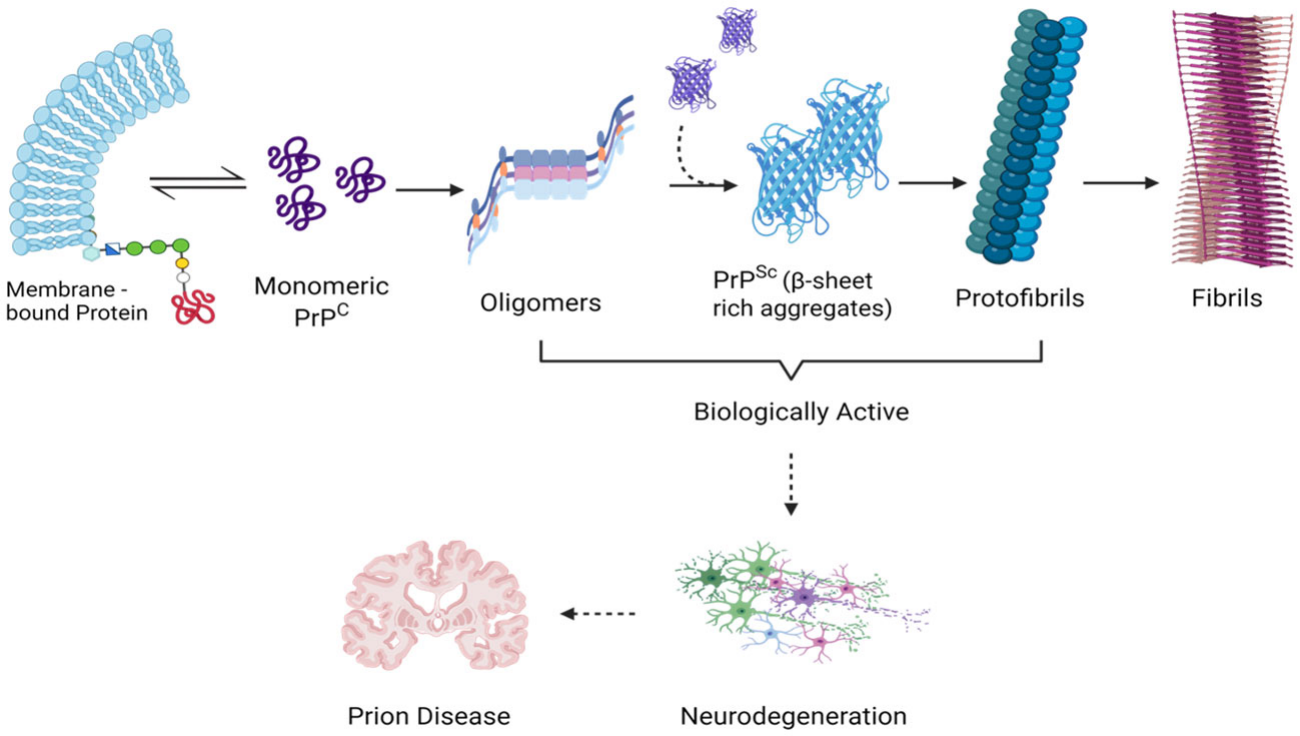
Demonstrating the Scientific Impact of Transitional Entities During Prion Aggregation: Analyzing Molecular Complexity. This diagram that illustrates the phases of prion aggregation emphasizes the pathogenicity linked to monomeric PrP^Sc^, small oligomeric assemblies, and PrP pre-fibrillar structures-all of which are thought to act as triggers for prion-induced neuronal death. Created with BioRender.com.

From a molecular perspective, the neurotoxic effects are triggered by small oligomeric structures following their internalization into neurons and subsequent accumulation within the endolysosomal compartment (Cascella et al., 2022). These aggregates are causally linked to lysosomal impairment, the release of proteolytic enzymes, and the activation of caspase-dependent apoptotic pathways (Audano et al., 2018). The cumulative experimental evidence robustly supports the proposition that the neurotoxicity observed in prion diseases predominantly arises from misfolded protein oligomers, constituting the primary initiators of the pro-apoptotic processes, in contrast to the larger fibrillar aggregates (**Figure 3**) (Lin et al., 2019; Drobny et al., 2023).

The human microbiome alludes to the diverse collection of microorganisms, including bacteria, viruses, fungi, and other microbes, that inhabit various parts of the body, including the gastrointestinal tract (Ursell et al., 2012). In the case of Creutzfeldt-Jakob disease (CJD), a common form of human prion disease, patients displayed gut microbiota alterations, with increased levels of actinobacteria, fusobacteria, and proteobacteria, and reduced firmicutes compared to their healthy counterparts (Guo et al., 2022). Recent research has shed light on possible associations between the gut microbiome and the development and progression of prion diseases, which involve the abnormal folding and clustering of the prion protein (D'Argenio and Sarnataro, 2019; Yang et al., 2020; Trichka and Zou, 2021; Guo et al., 2022). One aspect of prion disease pathogenesis that has received attention is the potential involvement of the gut-brain axis. The gut-brain axis is a complex bidirectional communication system between the gut and the central nervous system (CNS), involving neural, endocrine, and immune pathways. The gut microbiota exerts effects on prion disease via the gut-brain axis through the microglia activation, as a key component of the gut-brain axis, can influence CNS function through various mechanisms, including the production of neurotransmitters, modulation of the immune system, and regulation of inflammation (D'Argenio and Sarnataro, 2019; Mossad and Erny, 2020; Rutsch et al., 2020; Doroszkiewicz et al., 2021; Khatoon et al., 2023).

The gut microbiota produces diverse neurotransmitters and neuromodulators, including short-chain fatty acids (SCFAs), biogenic amines like histamine, and amino-acid-derived metabolites such as serotonin or GABA. The role of Short-Chain Fatty Acids (SCFAs) and common amino acids is crucial for promoting host neuroactive functions, particularly inflammatory phenotypes (microglia mediation), and neurotransmitter function in the neural system (Yang et al., 2020; Bairamian et al., 2022; O'Riordan et al., 2022). In patients with prion disease, SCFAs experience a substantial decrease due to the under-representation of *Prevotellaceae*, comprising acetate, propionate, and butyric acid (Guo et al., 2022; Salim et al., 2023). SCFAs play a role in connecting to the gut-brain axis, thereby regulating neural function. Bacterial products or metabolites from gut commensals, like SCFAs, may translocate from the intestinal mucosa to the systemic circulation, potentially interfering with immune regulation and central nervous system (CNS) function (Silva et al., 2020). SCFAs are generated through the fermentation of dietary carbohydrates and exhibit immunomodulatory properties (Parada Venegas et al., 2019; Liu et al., 2021; Ranjbar et al., 2021; Portincasa et al., 2022; Zhang et al., 2023).

The gut microbiome has been implicated in neuroinflammation, which is a prominent feature of prion diseases (D'Argenio and Sarnataro, 2019; Zhu et al., 2020; Li et al., 2021; Trichka and Zou, 2021). The postulated conjecture posits that prion agents ingested through dietary intake may instigate perturbations in the gut microbiota, leading to dysbiosis (D'Argenio and Sarnataro, 2019; Andrea et al., 2023; Ray et al., 2023). Subsequently, this dysbiotic state may elicit the generation of a microbial form of amyloid, eliciting an immune response that amplifies microglial and astrocytic activation in the brain. This, in turn, augments the production and deposition of neuronal amyloid in cerebral tissues. The purported interplay between ingested prions, gut dysbiosis, and cerebral amyloidosis offers insights into potential links between dietary factors and neurodegenerative pathogenesis. Perturbations in gut microbiome composition can incite immune responses, intensifying neuroinflammatory processes in prion pathology (Cerovic et al., 2019; Trichka and Zou, 2021; van Olst et al., 2021; Bostick et al., 2022).

Bacterial lipopolysaccharide (LPS) strongly activates microglial cells via the TLR4 pathway, leading to rapid inflammatory responses and the release of pro-inflammatory cytokines like IL-6 and TNF-α (Rosadini and Kagan, 2017; Batista et al., 2019; Yang et al., 2020; Skrzypczak-Wiercioch and Salat, 2022). Furthermore, bacterial enzymes have the ability to produce neurotoxic metabolites like as ammonia and d-lactic acid (Galland, 2014). D-lactic acid is mostly cleared by the kidneys and liver but is also produced by a variety of commensal gut microbes, most notably Lactobacillus and Bifidobacterium (Vitetta et al., 2017; Yilmaz et al., 2018; Remund et al., 2023). It is important to remember that the monocarboxylate transporter 1 (MCT1) allows a little amount of D-lactic acid to cross the blood-brain barrier (BBB). Raise levels of D-lactic acid in the brain have been linked to encephalopathy and concomitant suppression of neuronal uptake of L-lactic acid, resulting in cognitive deficits that could be related to prion disorders (Hanstock et al., 2010; Chen et al., 2022). The pivotal role of the gut microbiota in CNS development, function, and the pathophysiology of chronic brain diseases underscores the potent pro-inflammatory and innate-immune activation exerted by microbiome species and their secretory products in the host.

Numerous studies have been conducted to investigate the influence of the gut microbiome on prion disease progression, utilizing animal models (Bradford et al., 2017; D'Argenio and Sarnataro, 2019; Yang et al., 2020; Trichka and Zou, 2021; Khatoon et al., 2023). These studies have revealed that modifications in gut microbiota composition can impact prion disease susceptibility, incubation period, and severity of clinical symptoms. For instance, germ-free mice, lacking normal gut microbiota, demonstrated delayed onset and reduced severity of prion disease in comparison to conventionally raised mice (Bradford et al., 2017). Furthermore, studies involving the transfer of gut microbiota from prion-infected animals to germ-free mice have demonstrated the transmission of disease susceptibility (Trichka and Zou, 2021). However, Bradford et al. recently reported that the absence of commensal microbiota in germ-free mice did not affect prion disease duration or susceptibility following intraperitoneal or intracerebral injection of mouse-passaged 22C scrapie prions (Weissmann et al., 2011; Bradford et al., 2017).

Additionally, this study observed no differences in the magnitude and distribution of prion-characteristic neuropathological changes, including spongiform degeneration, accumulation of PrP^Sc^, astrogliosis, and microglial activation in the brain between conventional and germ-free mice.

## Unraveling the significance of the gut microbial on the pathogenesis of Parkinson’s disease

**Figure 4:**
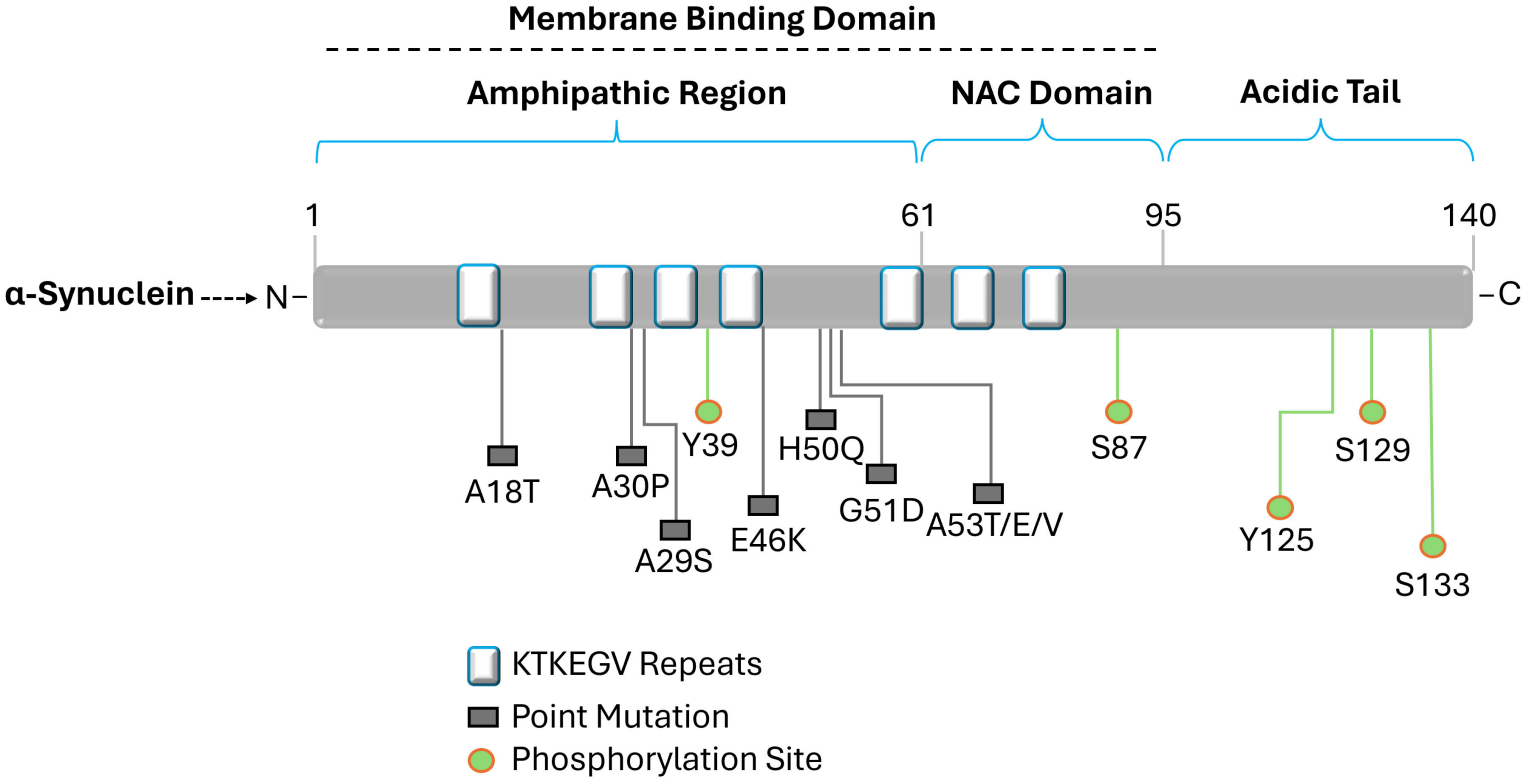
Diagram illustrating the structure of alpha-synuclein. Labeled amino acid residues include known sites of mutations and define the N-terminus, NAC region, and C-terminus. Three different domains can be differentiated from the 140 amino acid protein. The amino acid residues impacted by the primary alpha-synuclein gene mutations (A18T, A30P, A29S, E46K, H50Q, G51D, and A53T/E/V) linked to autosomal dominant Parkinson's disease are found in the N-terminal amphipathic domain. Membrane binding is carried out by the N-terminal region, which has a predisposition for helical folding. Aggregation is encouraged by the hydrophobic non-amyloid β-component of plaque (NAC) domain. The primary phosphorylation site is located at Ser129 in the acidic tail that the C-terminal domain produces and α-synuclein aggregation is modulated by the C-terminal domain.

The loss of dopamine-producing neurons in the substantia nigra and an accumulation of aberrant α-synuclein protein aggregates are the hallmarks of Parkinson’s disease, a complicated neurodegenerative condition (Eriksen et al., 2003; Stefanis, 2012; Meade et al., 2019; Gomez-Benito et al., 2020; Srinivasan et al., 2021; Wise et al., 2022). Although the exact etiology of Parkinson’s disease is still unknown, a mix of environmental and genetic variables are believed to be involved. Meanwhile, α-synuclein is a naturally occurring protein found abundantly in healthy nerve cells, especially in presynaptic terminals, where it plays a role in regulating synaptic vesicle function ad neurotransmitter release (Stefanis, 2012; Mor et al., 2016; Huang et al., 2019; Carnazza et al., 2022). It has a molecular weight of about 14 kDa and 140 amino acids. Interestingly, its structure lacks a stable, three-dimensional form and is inherently chaotic. The N-terminus, middle region, and C-terminus are its three primary structural features (Lashuel et al., 2013; Vaikath et al., 2022). The N-terminus engages in interactions with cellular membranes as well as other substances. Hydrophobic amino acids are present in the middle region, which contributes to the protein’s propensity to aggregate, an important feature in the pathophysiology of illness. Interactions between the C-terminus and cellular membranes are regulated (**Figure 4**) (Snead and Eliezer, 2014; Allen Reish and Standaert, 2015; Yeboah et al., 2019; Bisi et al., 2021; Liu et al., 2021; Calabresi et al., 2023).

Nevertheless, pathogenic alterations in α-synuclein occur in Parkinson’s disease, which furthers the disease’s advancement (Stefanis, 2012; Xu and Pu, 2016; Calabresi et al., 2023). Despite this, α-synuclein misfolds in Parkinson’s disease (PD), changing from its normally soluble form to an insoluble, aggregated form (Sharon et al., 2003; Breydo et al., 2012; Stefanis, 2012; Mehra et al., 2019; Vidovic and Rikalovic, 2022). Lewy bodies and Lewy neurites are the names given to these aggregates (Colom-Cadena et al., 2017; Mahul-Mellier et al., 2020). These aberrant protein deposits are thought to contribute to neuronal death by interfering with regular cellular processes. Furthermore, α-synuclein aggregates, toxic to neurons, cause their degeneration and eventual death (Cookson, 2009; Kim et al., 2023; Neupane et al., 2023).

**Figure 5:**
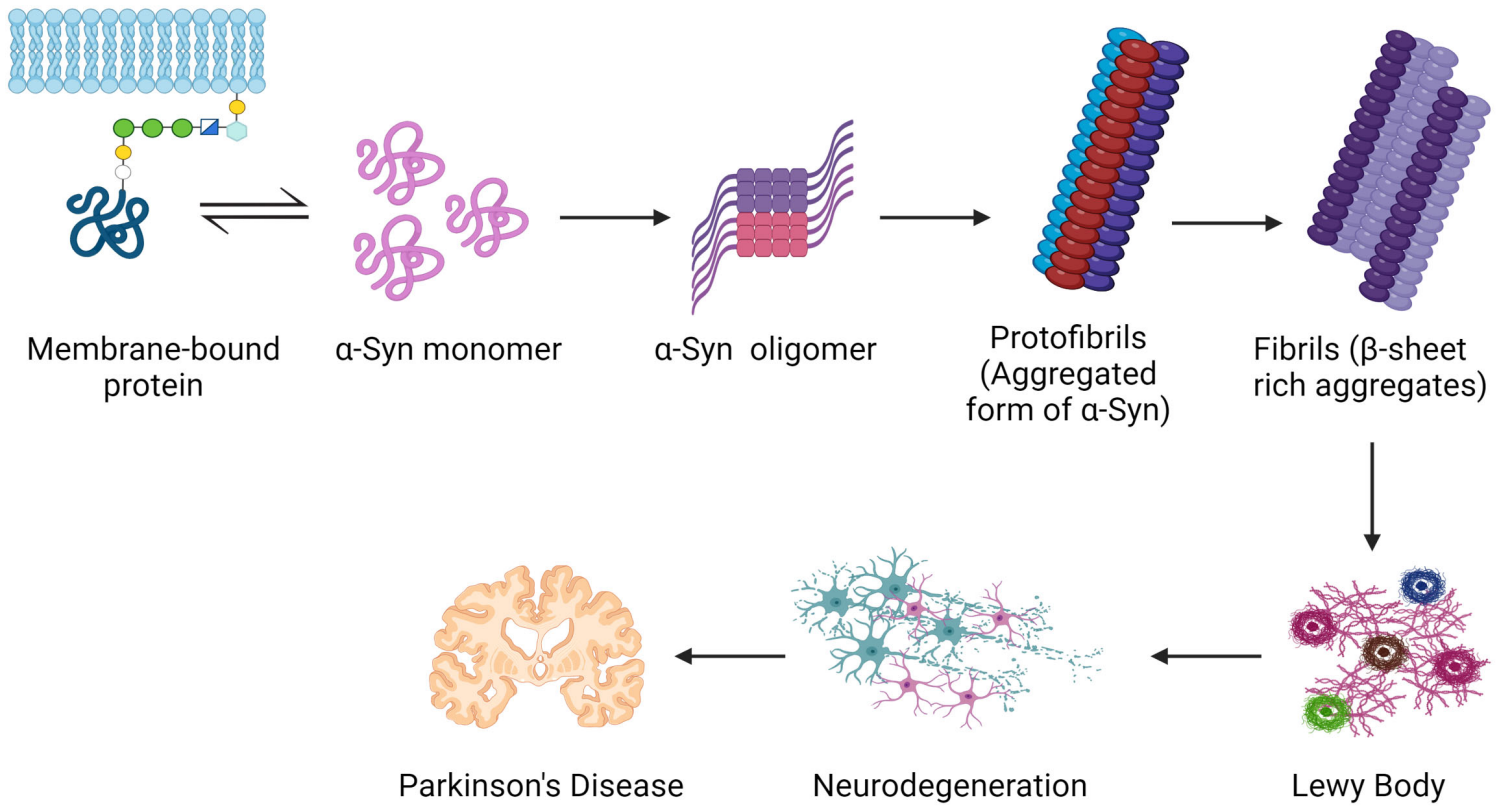
Common mechanisms serve as the groundwork for Parkinson’s disease pathogenesis. Inside nerve cells, the protein α-synuclein misfolds and creates poisonous clumps called Lewy bodies. The apparent motor symptoms of the illness might be driven by these aggregates, which can disrupt neuronal activity, impair cells, and ultimately perish dopamine-producing neurons. Created with BioRender.com.

The loss of dopamine-producing neurons in the substantia nigra causes a deficiency of dopamine in the brain because these neurons are especially vulnerable to this toxicity (Surmeier, 2018; Zhou et al., 2023). The underlying cause of Parkinson’s disease (PD) motor symptoms, such as tremors, bradykinesia, stiffness, and postural instability, is a dopamine deficit (DeMaagd and Philip, 2015; Varadi, 2020). Abnormal α-synuclein seeds are thought to have the ability to transfer from one neuron to another, aiding in the illness’s progression (**Figure 5**) (Angot et al., 2012; Chung et al., 2019; Henderson et al., 2019; Gomez-Benito et al., 2020).

Additionally, α-synuclein aggregation may be influenced by specific gut microbial populations, according to new research (Sampson et al., 2020; Faruqui et al., 2021; Nie et al., 2022; Singh et al., 2023). It is noteworthy that research has shown how vital volatile short-chain fatty acids (SCFAs), especially butyrate, are to preserving the integrity of the intestinal barrier (Parada Venegas et al., 2019; Silva et al., 2020; Liu et al., 2021; Perez-Reytor et al., 2021; Ma et al., 2022). Consequently, α-synuclein can be translocated from the stomach to the brain more easily when there is a shortage in SCFAs, which can result in increased intestinal permeability and the pathological spread of the protein (Kujawska and Jodynis-Liebert, 2018; Schaeffer et al., 2020; Lei et al., 2021; Chen and Lin, 2022; Singh et al., 2023).

Investigations into the gut microbiota of PD patients have revealed a significant reduction in the abundance of butyrate-producing bacteria, such as *Blautia*, *Coprococcus*, and *Roseburia*, in their fecal samples (Bullich et al., 2019; Shen, 2020; Heravi et al., 2023; Proano et al., 2023). Conversely, the mucosal-associated bacterial populations of healthy control subjects exhibit a richness in *Coprobacillaceae* (family), *Dorea* (genus), and the anti-inflammatory genus *Faecalibacterium* (Lei et al., 2021). Furthermore, *Prevotellaceae*, known to be involved in intestinal mucin formation and SCFA production through fiber fermentation in the sigmoid, shows decreased levels in the intestines of PD patients (Caputi and Giron, 2018; Bullich et al., 2019; Silva et al., 2020; Singh et al., 2023). This reduction in *Prevotellaceae* can lead to a decrease in intestinal mucus and an increase in intestinal permeability, facilitating the entry of α-synuclein into the enteric nervous system (ENS) via the intestinal barrier (Houser and Tansey, 2017; Lei et al., 2021). Consequently, this may contribute to the sustained expression of excessive α-synuclein or even promote its misfolding.

**Figure 6:**
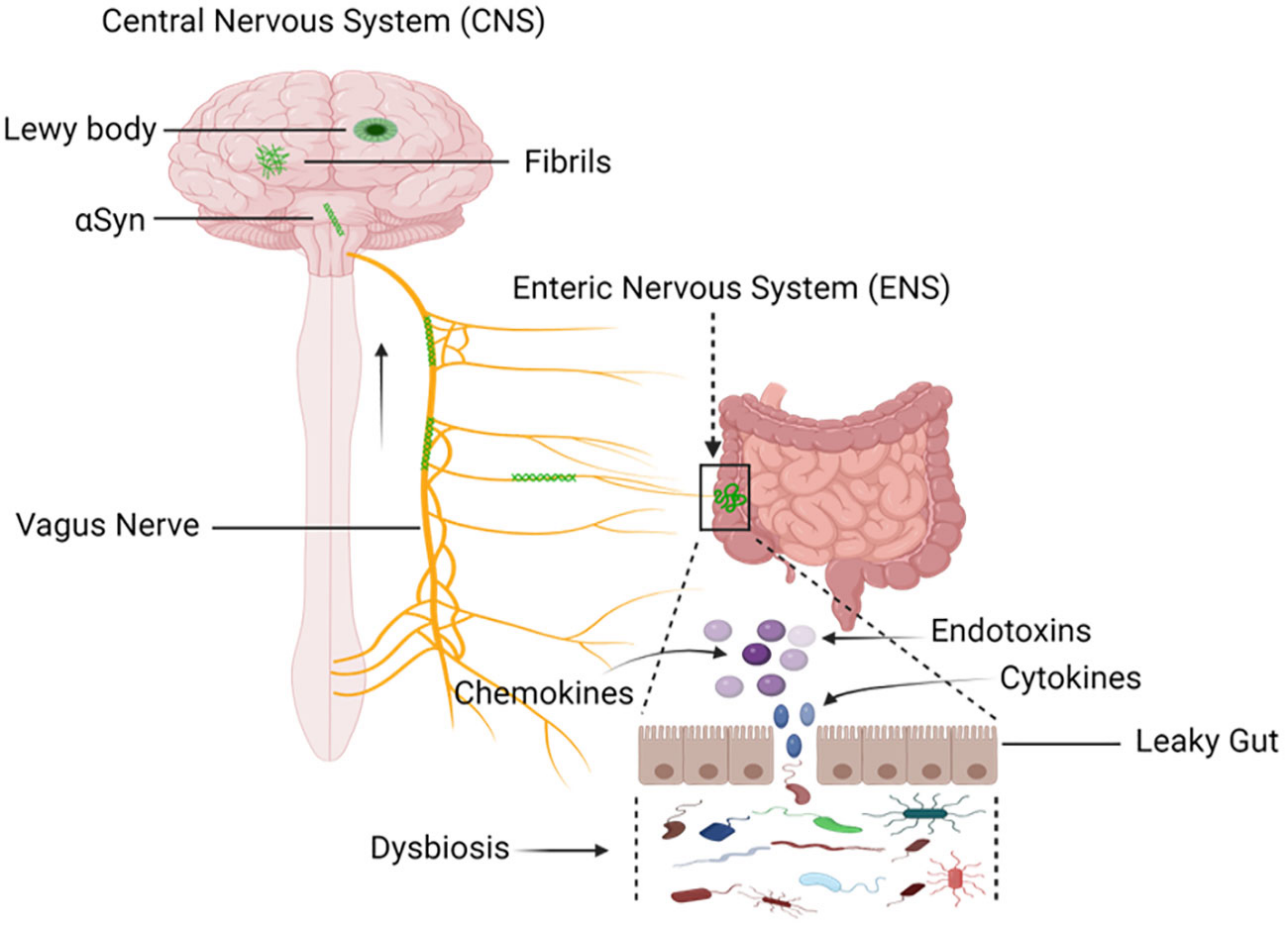
Mapping the Progression: Sequential Buildup and Transmission of α-Synuclein from Enteric Nervous System (ENS) to the Central Nervous System (CNS). Environmental factors, including microorganisms and the gastrointestinal microbiota (GM), induce a progressive buildup of α-synuclein within the extracellular matrix. Through this process, a pathological cascade that causes oxidative stress and mucosal inflammation is started, which eventually leads to the formation of α-synuclein aggregates. The vagal nerve is the proposed conduit. Pathological transmission of α-synuclein proceeds via the brainstem, midbrain, and basal forebrain, ultimately reaching cortical areas. The dynamics of α-synuclein pathogenesis from the ENS to the CNS are described in this visual representation. Created with BioRender.com.

The gut epithelium functions as a protective barrier against pathogen invasion. Disruption of gastrointestinal barriers can trigger a series of positive feedback loops that significantly alter the gut microbiota in favor of pro-inflammatory species, leading to intestinal inflammation and an increase in reactive oxygen/nitrogen species in the gut lumen (Jones et al., 2012; Kelly et al., 2015; Lin et al., 2022; Stolfi et al., 2022). This results in heightened mucosal permeability, oxidative stress, and inflammatory responses, along with the aggregation of α-synuclein in the enteric nervous system (ENS) (**Figure 6**) (Forsyth et al., 2011; Hirayama et al., 2023).

In a low-dose, oral rotenone-induced Parkinson’s disease mouse model, chronic stress-induced intestinal hyper-permeability and dysbiosis of gut microbiota promote the release of pro-inflammatory substances in the gut, leading to peripheral (substantia nigra) endotoxemia, neuroinflammation, and neurodegeneration (Gorecki et al., 2019; Li et al., 2019; Dodiya et al., 2020; Hamamah et al., 2022).

Commensal bacteria produce formylated peptides that bind to G protein-coupled receptors (GPCRs) on immune cells like macrophages and neutrophils, triggering inflammation in the gut epithelial cells (Jeong and Bae, 2020; Liang et al., 2020). This process results in the production of superoxide by NOX-1, leading to increased cellular reactive oxygen species (ROS) levels (Checa and Aran, 2020). *Lactobacilli* and *Bifidobacterium* in the gut can convert nitrate and nitrites into nitric oxide (NO), which can have neuroprotective effects in low concentrations and act as a neurotransmitter for noradrenergic, noncholinergic enteric neurons (Tiso and Schechter, 2015a; Tiso and Schechter, 2015b; Shandilya et al., 2022). However, at higher concentrations, NO leads to the detrimental production of reactive oxygen and nitrogen species (RONS) like superoxide and H_2_O_2_, contributing to neuroinflammation, axonal degeneration, and Parkinson’s disease pathogenesis (Yuste et al., 2015; Leyane et al., 2022).

An altered gut microbiome can shift the gut from a semi-permeable state to a hyper-permeable condition, commonly referred to as a “leaky gut.” This increased permeability allows microbial products, such as lipopolysaccharides (LPS), to enter the systemic circulation.

LPS is a potent inducer of inflammation, contributing to increased accumulation of α-synuclein and microglial activation, leading to the release of harmful substances that can damage neurons (Batista et al., 2019; Deng et al., 2020; Kwon and Koh, 2020; Bido et al., 2021; Skrzypczak-Wiercioch and Salat, 2022). LPS of gram-negative bacteria such *E. coli*, *Pseudomonas aeruginosa*, *Klebsiella pneumonia*, *Helicobacter pylori* etc. activate the toll like receptors 4 (TLR4) which are involve in recognition of bacterial pathogens, initiate a cascade of immune responses promoting production of pro-inflammatory cytokines, chemokines and oxidative factors (Albiger et al., 2007; Munford, 2008; Oliveira-Nascimento et al., 2012; Khan et al., 2016; Taban et al., 2022). Blood endotoxins raise blood levels of pro-inflammatory cytokines, and inflammation stimulates the blood-brain barrier (BBB) and the circumventricular organs (CVO), bringing leucocytes into the brain and raising brain cytokines that stimulate microglia, which causes loss of synapses and neurons (Harry and McPherson, 2014; Pathak and Sriram, 2023). Neuronal damage can initiate a process known as reactive microgliosis, ultimately resulting in the progressive degeneration of dopaminergic neurons (Tansey et al., 2007; Lull and Block, 2010).

The gut microbiome has been implicated in modulating neurotransmitter systems, including dopamine and serotonin, by producing and metabolizing neurotransmitters or their precursors (Strandwitz, 2018; Chen et al., 2021; Barandouzi et al., 2022; Dicks, 2022; Lynch and Hsiao, 2023; Miri et al., 2023). For instance, certain gut microbes can produce L-dopa, which can enter the brain through circulation and be converted into dopamine (Wang et al., 2021; Cirstea et al., 2023). Studies have examined the gut-brain communication activated by the effects of berberine (BBR) by transplanting *Enterococcus faecalis* or *Enterococcus faecium* into Parkinson’s disease (PD) mice (Wang et al., 2021; Hamamah et al., 2022; Carloni and Rescigno, 2023; Mitra et al., 2023). These bacteria significantly increased brain dopamine levels and improved PD symptoms in mice (Villageliu and Lyte, 2018). Moreover, combining BBR with the bacteria showed superior therapeutic effects compared to using bacteria alone (Cheng et al., 2022). Several neurotransmitters that are important for controlling social behavior, including glutamate, γ-aminobutyric acid (GABA), norepinephrine (NE), dopamine, and serotonin (5-HT), are either expressed or regulated by gut flora (Santos et al., 2019).

Interestingly, under the influence of the gut microbiota, enterochromaffin cells (EC) in the gut create a substantial amount of the body’s serotonin (5-HT) (Santos et al., 2019). Although changes in serotonin synthesis in the gastrointestinal (GI) tract may not directly impact the central nervous system (Camilleri, 2009; Terry and Margolis, 2017), alterations in the gut microbial composition can disturb the balance of these neurotransmitters, potentially influencing motor control, mood, and cognitive function, all of which are affected in PD (Silva et al., 2020; Chen et al., 2021).

A complicated interaction between the microbial community and host cellular processes has been established, with dysbiosis within the gut microbiome linked to the start of mitochondrial dysfunction and increased oxidative stress (Clark and Mach, 2017; Imdad et al., 2022; Zhang et al., 2022). The evolutionary origins of mitochondria from *alphaproteobacteria* emphasize this connection and show how closely related these organelles are to bacteria (Carvalho et al., 2015). Dysbiosis can affect a system in two ways: it can cause the generation of toxic metabolites and disrupt the host’s energy metabolism (DeGruttola et al., 2016). For instance, *Clostridium difficile’s* toxin B suppresses the Rho GTPases signaling system, changing the potential of the mitochondrial membrane, and jeopardizing the intestinal epithelial barrier at the same time (Chen et al., 2015; Chidambaram et al., 2022; Liu et al., 2022). This combined effect makes it easier for viruses and harmful substances to pass through the epithelium and reach the enteric nerve system (ENS), which could be detrimental to the digestive tract’s overall health.

In turn, the intestinal barrier’s weakened state permits harmful substances to enter the ENS without restriction (Ghosh et al., 2020). The vagus nerve, which mediates the complex gut-brain axis, is the essential conduit between the ENS and the central nervous system (Breit et al., 2018). This communication channel becomes a channel for signals, such as those pertaining to oxidative stress and mitochondrial function. The gut, especially the colon where the majority of gut microbiota resides, is coated with a layer of sticky mucus that aids in shielding the gut-blood barrier from pathogen invasion (Carlson et al., 2018; Fang et al., 2021; Gierynska et al., 2022). However, for this protective system to function effectively, it requires an adequate supply of energy, primarily provided by mitochondria. If mitochondria fail to deliver the required energy for the immune system, an unhealthy shift in the gut microbiota occurs, known as dysbiosis (Kramer, 2021; Casanova et al., 2023). As individuals age, both mitochondria and the microbiota deteriorate, providing gut pathogens multiple opportunities to disrupt brain function, either indirectly or directly (Vezza et al., 2020; Kramer, 2021; Borbolis et al., 2023; Righetto et al., 2023). In the realm of Parkinson’s disease investigations and other mental disorders, mitochondrial dysfunction, dysbiosis, and intestinal disease often occur both in conjunction with and preceding these conditions. Within the broader context of neurodegenerative illnesses, oxidative stress and mitochondrial dysfunction become key players in the pathophysiology, particularly in Parkinson’s disease (PD) (Guo et al., 2013; Hauser and Hastings, 2013; Bhat et al., 2015; Henrich et al., 2023). This supports the hypothesis that disturbances in the gut microbiota, which affect mitochondrial health and oxidative balance, could have a role in the onset or aggravation of diseases such as Parkinson’s disease (PD) via way of the complex gut-brain axis network.

The gut microbiome communicates bidirectionally with the brain through the gut-brain axis, facilitated mainly by afferent and efferent fibers of the vagus nerve (Carabotti et al., 2015; Bonaz et al., 2018; Longo et al., 2023). This neural pathway serves as a direct route for signals and molecules produced by the gut microbiome to reach the brain (Carabotti et al., 2015; Martin et al., 2018). These signals have the potential to influence neurotransmission, neuroinflammation, and neural plasticity, all of which are involved in the pathogenesis of Parkinson’s disease (PD).

Several preclinical studies have indeed suggested a crucial role of the vagus nerve in the gut-brain axis (Dinan and Cryan, 2017; Benakis and Liesz, 2022; Han et al., 2022). Vagotomy, which involves cutting or inhibiting the vagus nerve, has been shown to block brain neurotoxicity in certain animal models (Liu and Forsythe, 2021). This implies that the vagus nerve may play a role in transmitting signals between the gut and the brain that can influence neurological health. Furthermore, vagus nerve stimulation has been explored as a potential therapeutic approach for various conditions, including Parkinson’s disease (Farrand et al., 2017; Farrand et al., 2019; Jin et al., 2023). The vagus nerve stimulation involves the use of electrical impulses to stimulate the vagus nerve, and it has been investigated for its potential benefits in modulating neural activity and potentially alleviating symptoms of certain neurological disorders (Howland, 2014; Capilupi et al., 2020; Yap et al., 2020; Fang et al., 2023).

The gut microbiota has the capacity to produce a diverse array of molecules, including neurotransmitters, neuropeptides, and metabolites, which can act as signaling molecules impacting neurotransmission in the brain (Chen et al., 2021; Miri et al., 2023). For instance, certain gut bacteria such as *Morganella morganii, Klebsiella pneumoniae*, and *Hafnia alvei* are capable of producing neurotransmitters like dopamine, which play crucial roles in various brain functions, including movement control, motivation, reward, and mood regulation (Strandwitz, 2018; Averina et al., 2020; Dicks, 2022).

Nevertheless, environmental factors, such as dietary choices, exposure to toxins, and antibiotic use, can influence the composition and function of the gut microbiome, potentially impacting PD pathogenesis (Hasan and Yang, 2019; Zheng et al., 2020; Salim et al., 2023). The Western diet, characterized by high caloric intake, saturated and omega-6 (ω6) fatty acids, refined sugars, excessive salt, and low consumption of omega-3 (ω3) fatty acids and fiber, is considered a risk factor for PD (Jackson et al., 2019; Zapala et al., 2022). This diet adversely affects the beneficial microbiome. On the other hand, adhering to a Mediterranean diet, which includes fresh vegetables, fruits, nuts, seeds, non-fried fish, olive oil, wine, coconut oil, herbs, and spices, has been linked to a lower chance of acquiring Parkinson’s disease (Mischley et al., 2017; Bisaglia, 2022).

Research has demonstrated that prolonged exposure to broad-spectrum antibiotics can eliminate beneficial microorganisms, leading to alterations in intestinal permeability and an increased risk of PD (Ramirez et al., 2020; Karakan et al., 2021; Varesi et al., 2022; Salim et al., 2023). Antibiotics, originally designed for bacterial infections, have garnered attention for their potential neuroprotective properties in neurodegenerative disorders (Yadav et al., 2021). Their anti-inflammatory, immunomodulatory, and anti-amyloidogenic effects, coupled with antioxidant capabilities, extend beyond their antimicrobial role (Nadeem, 2006). In the context of 1-methyl-4-phenyl-1,2,3,6-tetrahydropyridine (MPTP)-induced Parkinson’s disease (PD) in mice, the preservation of tyrosine hydroxylase (TH) immunoreactivities in the substantia nigra and dopamine transporter (DAT) immunoreactivities in the striatum, typically compromised by MPTP, was achieved through treatment with a combination of broad-spectrum antibiotics (ampicillin, metronidazole, and neomycin sulfate) (Pu et al., 2019; Fang et al., 2020; Varesi et al., 2022). This positive outcome was associated with an increase in *Proteobacteria* and a decrease in *Deferribacteres* and *Saccharibacteria* (TM7) abundance, indicative of an altered gut microbiota composition characterized by reduced diversity (Pu et al., 2019; Varesi et al., 2022). The findings of this study suggest that antibiotic-induced microbiome depletion may confer protection against MPTP-induced dopaminergic neurotoxicity in the mouse brain. Additionally, MPTP exposure appeared to positively influence the diversity and composition of the gut microbiota in antibiotic-treated mice (Koutzoumis et al., 2020; Shan et al., 2021). In a recent study by Cui et al., it was revealed that pretreating MPTP-induced Parkinson’s disease (PD) mice with vancomycin led to notable improvement in motor symptoms (Cui et al., 2023). This improvement was associated with a decrease in astrocyte and microglia activation in the substantia nigra (SN) (Koutzoumis et al., 2020; Cui et al., 2023). The authors posited that the increased presence of *Akkermansia* and *Blautia*, induced by vancomycin, played a crucial role in indirectly mitigating neuroinflammation. This mitigation was achieved through interference with the toll-like receptor 4 (TLR-4)/NF-κB pathway, impacting both the gut and the brain (Varesi et al., 2022; Cui et al., 2023).

Similarly, Rats induced with Parkinson’s disease (PD) using 6-OHDA and undergoing prolonged antibiotic treatment (neomycin, pimaricin, bacitracin, and vancomycin) exhibited results akin to those observed in previous studies. The intervention forestalled dopaminergic neuronal demise, alleviated inflammation, improved neurotoxicity, and lessened motor impairments, as determined by cylinder, rotation, and stepping tests (Varesi et al., 2022; Cui et al., 2023). These findings underscore the importance of the gut microbiota in the development of Parkinson’s disease, with various factors collectively contributing to a distinctive pattern associated with the disease.

## Conclusion

In a nutshell this comprehensive analysis highlights the complex and multidimensional relationship between the pathophysiology of neurodegenerative illnesses and the gut microbiota, with particular attention to Parkinson’s disease and prion diseases. Due to its involvement in neuroinflammation, modulation of neurotransmitters, mitochondrial function, and preservation of intestinal barrier integrity through the intricate gut-brain axis, the gut microbiota is crucial for both the onset and progression of numerous detrimental neurological disorders, especially prion diseases and Parkinson’s disease (PD).

The composition of the gut microbiota and, thus, the vulnerability to Parkinson’s disease and prion diseases, are significantly influenced by dysbiosis, dietary components, and environmental variables. Moreover, these facts have translational significance as demonstrated by the strong results obtained from clinical studies and animal models. Moreover, the molecular mechanisms explored in this study have emphasized the significance of microbial metabolites, such as bacterial lipopolysaccharides (LPS) and short-chain fatty acids (SCFAs), which are generated by the gut microbiome, in regulating brain functioning as well as having a severe influence on Parkinson’s disease and prion diseases, and underscored their potential as an intriguing area of study for comprehending the complex interrelationships between the gut and the brain.

Even though more investigation is required to clarify the exact causal pathways and connections underlying these occurrences, the data provided here provides a strong basis for future studies on the gut-brain axis in relation to Parkinson’s disease and prion diseases. Understanding and utilizing the influence of the gut microbiota on brain health could lead to the creation of novel therapeutic and preventive measures, providing hope to those suffering from these difficult and debilitating neurodegenerative disorders.

## Author contributions

NM: Conceptualization, Validation, Visualization, Writing – original draft, Writing – review & editing. MI: Conceptualization, Validation, Visualization, Writing – original draft, Writing – review & editing. SH: Conceptualization, Funding acquisition, Project administration, Resources, Supervision, Writing – review & editing. HC: Conceptualization, Funding acquisition, Resources, Supervision, Validation, Writing – review & editing.
